# Whole-Genome Assemblies for Three Yersinia pestis Strains Isolated in Erenhot, China

**DOI:** 10.1128/MRA.01084-20

**Published:** 2020-11-05

**Authors:** Jing Wang, Xifeng Yang, Hongyuan Zheng, Li Tian, Qi Shi, Jing Wu, Yujun Cui, Yajun Song, Wenxiu Guo, Tuoya Yun, Hua Yun, Muzi Jin, Yufei Chen, Sheng Zhang, Huaibo Wei

**Affiliations:** a Chinese Academy of Inspection and Quarantine (CAIQ), Beijing, China; b Huhehaote Customs District P.R. China, Hohhot, China; c State Key Laboratory of Pathogen and Biosecurity, Beijing Institute of Microbiology and Epidemiology, Beijing, China; University of Maryland School of Medicine

## Abstract

To explore the genetic diversity of Yersinia pestis strains in Erenhot, China, and their relationship with Mongolian strains, we collected and sequenced three Y. pestis strains from Erenhot, China, in 2018. Here, we report the draft genome sequences of three Y. pestis bv. Medievalis strains belonging to the 2.MED phylogroup that were circulating in Meriones unguiculatus populations.

## Abstract (precis)

To explore the genetic diversity of Yersinia pestis in Erenhot, China, and their relationship with Mongolia strains, we collected and sequenced three Y. pestis strains from Erenhot, China, in 2018. Here, we report the draft genome sequences of three Y. pestis bv. Medievalis strains belonging to the 2.MED phylogroup that were circulating in Meriones unguiculatus populations.

## ANNOUNCEMENT

Yersinia pestis, a Gram-negative bacterium that belongs to *Enterobacteriaceae*, is the pathogen that caused three major pandemics of plague which have claimed the lives of millions of people and have greatly influenced human history on a global scale (1). Through a calculation based on the neutral molecular clock, Y. pestis differentiated from its ancestor Yersinia pseudotuberculosis around 6,000 years ago, and Y. pestis strains could be attributed to five major branches according to whole-genome-wide variations (2, 3).

Inner Mongolia, located in northern China with an area of about 1.18 million km^2^, is an autonomous region of China bordering Mongolia to the north. Erenhot, which is under the pressure of plague every year like other plague foci, is a border city of Inner Mongolia connecting China and Mongolia. In this study, we isolated three Y. pestis strains from the carcass of a Mongolian gerbil (Meriones unguiculatus) and the fleas collected from the carcass in 2018. The carcass was found about 700 m south of the borderline on the Inner Mongolia side of the border (43.39 N 111.54 E). Strain S14 was isolated from the liver of the dead gerbil, and strains S15 and S16 were isolated from the Xenopsylla conformis and Nosopsyllus laeviceps fleas collected from the carcass, respectively. These three isolates were cultured on Hottinger’s agar (pH 6.9 to 7.1; Lanzhou Biological Products Research Institute Technology Co. Ltd.) at 28°C for 2 days, and their identity was confirmed with microscopy examination and Yersinia pestis bacteriophage lysis (Lanzhou Biological Products Research Institute Technology Co. Ltd.), PCR (rapid detection of Yersinia pestis and *Hantavirus* on rodents at frontier port; SN/T 2616-2010; standard of General Administration of Quality Supervision, Inspection and Quarantine, China), and colloidal gold immunochromatography (test kit for plague F1 antigen; Beijing Zhuangdi Haohe Bio-medical Technology Co. Ltd.) tests. Then, these three isolates were cultured on Hottinger’s agar for 18 h for DNA extraction, and DNA samples were extracted using the Qiagen DNeasy blood and tissue kit.

Sequencing libraries were prepared using the MGIEasy universal DNA library prep set kit (BGI, Shenzhen, China), and whole-genome sequencing (WGS) was performed using the Illumina NovaSeq 6000 platform according to the manufacturer’s instructions. For the Illumina sequencing library, the insert size was 350 bp, with a paired-end sequencing length of 150 bp. Finally, we obtained 32,736,224, 33,412,632, and 33,112,132 paired-end raw reads for strains S14, S15, and S16, respectively. Then, we used Trimmomatic v0.38 (4) to remove the low-quality sequencing reads (quality value [QV], >20). After filtering the raw data, we obtained 678 Mb, 915 Mb, and 604 Mb clean reads for each isolate, and the coverage for each genome is 145×, 196×, and 129×. The paired-end reads were *de novo* assembled by SPAdes v3.12.0 (5), and coding DNA sequences (CDSs) of the assembled genomes were annotated via NCBI PGAP v4.11 (6, 7). All the software settings used were under the default parameters unless otherwise mentioned. Finally, we obtained 220, 214, and 211 contigs for strains S14, S15, and S16, respectively. The genome characteristics of these strains are recorded in Table 1.

**TABLE 1:** Basic statistics on assemblies and annotations

Strain	BioSample accession no.	Size (bp)	Coverage (×)	No. of contigs	No. of CDSs	G+C content (%)	*N*_50_ (bp)
S14	SAMN16205172	4,681,226	145	220	4,106	47.54	45,390
S15	SAMN16205173	4,681,214	196	214	4,104	47.54	47,111
S16	SAMN16205174	4,681,619	129	211	4,104	47.54	46,925

### Data availability.

These whole-genome shotgun projects for strains S14, S15, and S16 have been deposited in DDBJ/ENA/GenBank under the accession numbers JACXWW000000000, JACXWV000000000, and JACXWU000000000, respectively. The versions described in this paper are the first versions, JACXWW000000000.1, JACXWV000000000.1, and JACXWU000000000.1. The Sequence Read Archive (SRA) data for strains S14, S15, and S16 have been deposited in the NCBI SRA under the accession numbers SRR12667592, SRR12667591, and SRR12667590, respectively.
